# Performance of the Colloidal Gold Immunochromatography of Cryptococcal Antigen on Bronchoalveolar Lavage Fluid for the Diagnosis of Pulmonary Cryptococcosis

**DOI:** 10.1155/2022/7876030

**Published:** 2022-07-09

**Authors:** Ning Zhu, Shanhong Lin, Xingbei Weng, Weijie Sun, Xueqin Chen

**Affiliations:** ^1^Department of Respiratory and Critical Care Medicine, Ningbo First Hospital, Ningbo, China; ^2^Department of Ultrasound, Ningbo First Hospital, Ningbo, China; ^3^Department of Laboratory Medicine, Ningbo First Hospital, Ningbo, China; ^4^Department of Traditional Medicine, Ningbo First Hospital, Ningbo, China

## Abstract

**Objective:**

This study aimed to investigate the efficacy of the colloidal gold immunochromatography method in the detection of *Cryptococcus* antigen in bronchoalveolar lavage fluid (BALF) for pulmonary cryptococcosis (PC) diagnosis.

**Methods:**

A total of 111 patients with clinically suspected PC who were finally diagnosed with nonhuman immunodeficiency virus infection and hospitalized in the Ningbo First Hospital from March 2017 to December 2021 were retrospectively analyzed. All the confirmed cases were divided into two groups as follows: the PC group (33 cases) and the non-PC group (78 cases). All the patients were subjected to serum and BALF cryptococcal capsular polysaccharide antigen-lateral flow immunochromatographic assay (CrAg-LFA) and etiological culturing.

**Results:**

In the PC group, serum CrAg-LFA was positive for 24 and negative for 9 cases, serum *Cryptococcus* culture was positive for 1 and negative for 32 cases, BALF CrAg-LFA was positive for 31 and negative for 2 cases, and BALF *Cryptococcus* culture was positive for 9 and negative for 24 cases. In the non-PC group, serum CrAg-LFA was positive for 1 and negative for 77 cases, serum culture was negative in all the cases, and both BALF CrAg-LFA and culture were negative in all the cases. The sensitivity, specificity, and accuracy of BALF CrAg-LFA for PC diagnosis were 93.9%, 100%, and 98.2%, respectively, whereas those of BALF culture were 27.3%, 100%, and 78.4%, respectively. The sensitivity and accuracy of BALF CrAg-LFA were higher than that of serum CrAg-LFA and BALF etiological culture with statistically significant differences (*p* < 0.05).

**Conclusion:**

The diagnostic value of BALF CrAg-LFA for PC is superior to that of serum CrAg-LFA and BALF etiological culture.

## 1. Introduction

Pulmonary cryptococcosis (PC) is a pulmonary fungal disease that can be acute, subacute, or chronic and is caused by inhaling *Cryptococcus* spores into the respiratory system. Recently, an increase in the incidence rate of PC has been observed. Although the disease is prevalent in immunocompromised patients, it is becoming increasingly common in immunocompetent patients as well [1]. PC is easily misdiagnosed or missed due to the absence of specific clinical symptoms and imaging findings. Since *Cryptococcus* locally infiltrates and infects the lung tissue [2], serum cryptococcal capsular polysaccharide antigen-lateral flow immunochromatographic assay (CrAg-LFA) is a better option for its detection due to its high sensitivity and specificity, simple operation, low-cost, speediness, and noninvasiveness, compared with traditional etiological investigations (ink staining and culturing) and pathology tests. In addition to the serum CrAg test and invasive histopathological investigation, a third diagnostic method deserves further study, especially for serum CrAg-negative patients. A study showed that compared with detection of galactomannan antigen (GM) in serum, its detection in bronchoalveolar lavage fluid (BALF) has a higher diagnostic value for mycosis [3]. However, to the best of our knowledge, there exists a dearth of studies regarding the efficacy of performing CrAg-LFA on BALF for PC diagnosis. In the present study, the effectiveness of the clinical diagnosis of PC by BALF CrAg-LFA was investigated by comparing the results of *Cryptococcus* antigen detection by CrAg-LFA in BALF and serum samples.

## 2. Materials and Methods

### 2.1. Clinical Data

A total of 111 patients with clinically suspected PC hospitalized in the Ningbo First Hospital from January 2017 to December 2021 were retrospectively analyzed. The inclusion criteria of suspected PC were patients with nonacquired immune deficiency syndrome with PC imaging features who were not confirmed by serology, etiology, or pathology during hospitalization and who agreed to receive bronchoscopy. Radiographic features of the suspected patients included single or multiple nodules and masses and localized or extensive pneumonia-like infiltration and consolidation. The exclusion criteria included patients who were extremely weak and unable to tolerate bronchoscopy, had a history of *Cryptococcus* infection, had severe cardiopulmonary insufficiency, showed difficulty in cooperation due to mental disorders, could not be accurately diagnosed after blood and BALF investigations, had lung puncture conditions but refused the investigation, were using antifungal treatment, and were pregnant.

33 patients were diagnosed with PC and nonhuman immunodeficiency virus (HIV) infection (PC group), including 23 who were pathologically confirmed by surgery or biopsy and 10 by positive BALF or blood culture. All PC patients were confirmed using (1) lung histopathologic evidence from surgical resection, bronchoscopy, or percutaneous lung biopsy, which was characterized by noncaseous granuloma with phagocytic *Cryptococcus* cells and cytoplasmic capsules of macrophages or (2) positive culture of *C. gattii* or *C. neoformans* from biopsy specimen, blood culture, or BALF [4, 5]. 78 patients were diagnosed as non-PC and included in the non-PC group. The final diagnosis of all non-PC patients was in accordance with the guidelines for the relevant diseases. This study was approved by the Ethics Committee of the Ningbo First Hospital (Approval Document No. 2021RS063).

### 2.2. Methods

#### 2.2.1. Instruments and Reagents

The flexible electronic bronchoscope (BF260, BF-1T260, BF–F260, OLYMPUS, Japan) and *Cryptococcus* antigen detection kit (colloidal gold method) manufactured by Immuno-Mycologic (USA), the API 20C AUX fungi identification system and the BacT/Alert 3D automatic hemoculture (blood culture) instrument purchased from BioMérieux, France, and the BACTEC FX automated hemoculture (blood culture) instrument purchased from BD Company, USA, were used.

#### 2.2.2. Sample Collection and Analysis

1. BALF sample collection: following hospital admission, the patients in each group were administered local anesthesia using lidocaine 2%. The tip of a fiber bronchoscope was tightly inserted into the opening site of the targeted subsegment bronchus. A total of 60 mL of sterile normal saline at 37°C was continuously and rapidly injected through the duct for lavage (20 mL/time, 2-3 times in total), following which 20–30 mL BALF was retrieved by suction under negative pressure of 13.30–9.95 kPa and centrifuged at low temperatures within 24 h to obtain the supernatant. The supernatant was then stored for testing.
2. CrAg-LFA: the *Cryptococcus* antigen detection kit (colloidal gold method, Immy Company) was used to detect serum and BALF *Cryptococcus* antigen according to the manufacturer's instructions regarding operation procedure and result interpretation.
3. *Cryptococcus* culture: after collection, the BALF sample was subjected to quantitative culturing, i.e., each 10 *μ*L of the sample was inoculated on blood plate, chocolate plate, and Sabouraud's medium. The remaining BALF was put into a 20 mL sterile test tube and centrifuged for 10 min at 3,000*g*. The supernatant was collected for CrAg detection, and the results were observed after 10 min. The supernatant was discarded and the BALF sediment was collected for further culture; a certain amount of BALF sediment was inoculated into blood plate, chocolate plate, and two Sabouraud's media. A portion of Sabouraud's media inoculated with BALF sediment was placed in an ordinary incubator at 25°C, while the rest of the media was placed in a 5% CO_2_ incubator at 35°C and observed once daily for 2 consecutive weeks. After the formation of colonies that were visible to the naked eye under the culture condition of 35°C within 2 weeks, the API 20CAUX identification system was used for the final identification. The supernatant samples were used for hemoculture (blood culture). Additionally, 5 mL of serum was collected under fasting conditions, and the supernatant was stored in a refrigerator at −20°C. The culture from the flasks with alarming positive growth was immediately transferred and inoculated in a blood agar plate and Sabouraud's plate. Following their growth and purification by isolation, the API 20CAUX identification system was used for their identification.

### 2.3. Statistical Analysis

The data were processed by using SPSS 22.0 statistical software. Measurement data conforming to normal distribution were expressed as mean standard deviation ($\bar{x}$ ± *s*) and enumeration data as percentages (%). Nonnormally distributed data were analyzed by using the Kruskal–Wallis test. The chi-square test (including the chi-squared test with continuity correction and McNemar test) or Fisher exact probability method was used for the intergroup comparison of categorical or ranked data. The difference was considered statistically significant with a *p* value of <0.05.

## 3. Results

### 3.1. Comparison of Demographic Characteristics and Clinical Data

Of the 111 patients, 33 were diagnosed with PC. More than half of the patients with PC (18 cases, 54.5%) were asymptomatic and presented with abnormal pulmonary imaging that was found during physical examination or investigation for other reasons. Cough or sputum was relatively common, whereas fever, shortness of breath, hemoptysis, and chest pain or distress were uncommon in patients with clinical symptoms. Preexisting diseases included chronic bronchitis, diabetes mellitus, cirrhosis, chronic kidney disease, malignant solid tumor, and autoimmune disease in the PC group, whereas 10 patients (30.3%) had no preexisting disease. No statistically significant difference was found among the immunodeficiency host (glucocorticoid or immunosuppressant treatment), cellular immune function (CD4^+^ or CD8^+^ T lymphocytes), and infection-related inflammation markers (white blood cells or C-reactive protein) between the two groups (*p* > 0.05). Additionally, chest CT showed a more frequent presence of multiple nodules or masses in the PC group compared with the non-PC group (33.3% vs. 15.4%, *p*=0.006) (Table 1).

### 3.2. Serum and BALF CrAg-LFA Test and Microbial Culture Results

Of the 33 patients with PC, serum cryptococcal culture was positive for only 1 (3.0%) and negative for 32 cases, whereas BALF culture was positive for 9 (27.3%) and negative for 24 cases. Significant differences were found in the etiological culture results between the PC and non-PC groups (*p* < 0.001) (Table 2). Additionally, in the PC group, serum CrAg-LFA was positive for 24 (72.7%) and negative for 9 (27.3%) cases, while BALF CrAg-LFA was positive for 31 (93.9%) and negative for only 2 (6.1%) cases. A significant difference was found between the results of serum and BALF CrAg-LFA in these two groups (*χ*^2^ = 4.90, *p*=0.021; *χ*^2^ = 0.50, *p*=0.049, respectively) (Table 3).

### 3.3. Comparison of BALF CrAg-LFA, Serum CrAg-LFA, and Microbial Culture for PC Diagnosis

The sensitivity, specificity, and accuracy of serum CrAg-LFA for PC diagnosis were 72.7%, 98.7%, and 91.0%, respectively, and that of BALF CrAg-LFA was 93.9%, 100%, and 98.2%, respectively. Both the sensitivity and accuracy of BALF CrAg-LFA for PC diagnosis were superior to those of serum CrAg-LFA, with a statistically significant difference (*χ*^2^ = 4.000, *p*=0.039; *χ*^2^ = 6.125, *p*=0.008, respectively) (Table 4). The sensitivity, specificity, and accuracy of BALF culture for PC diagnosis were 27.3%, 100%, and 78.4%, respectively. The sensitivity and accuracy of BALF culture were lower than those of BALF CrAg-LFA, with a statistically significant difference (*χ*^2^ = 20.045, *p* < 0.001; *χ*^2^ = 18.375, *p* < 0.001, respectively) (Table 5).

## 4. Discussion

The incidence of PC has been increasing each year. However, its clinical manifestations continue to lack specificity. This study revealed that more than half of the patients (18 cases, 54.5%) with PC were asymptomatic and 30.3% (10 cases) had no preexisting diseases. In addition to the main clinical manifestation of multiple nodules or masses, other manifestations were also present. The specificity of the chest CT for the detection of the clinical symptoms of PC was low, which was also found in previous studies [6–8], indicating how easy it was to misdiagnose or delay the diagnosis of PC, which further results in aggravated conditions. A good prognosis depends on early disease diagnoses and specific treatments. For PC, etiological and pathological investigations are the traditional diagnostic methods [9]; however, they remain nonconducive for early disease diagnosis due to being time-expensive and having low sensitivity, as well as because of the invasiveness of the pathological tests. Nevertheless, the recent and rapid development of detection techniques for *Cryptococcus* antigen makes early PC diagnosis possible. Present, the detection methods for serum cryptococcal capsular antigen primarily include latex agglutination, enzyme-linked immunosorbent assay (ELISA), and colloidal gold immunochromatography (CrAg-LFA) [10, 11]. Previous studies have revealed that CrAg-LFA is more accurate than latex agglutination and ELISA, possessing a wide prospect for clinical application with the advantages of simple operation, fast diagnosis, and fewer laboratory and operator requirements [12].

The colloidal gold method is a novel method for the fast diagnosis of cryptococcal infection and is also an immunochromatographic dipstick method for qualitative or semiquantitative antigen detection [13, 14]. The cryptococcal capsular polysaccharide test strip (colloidal gold method) is a simple and rapid detection reagent by which four serotypes (A–D) of *Cryptococcus neoformans* can be clearly and accurately detected using colloidal gold-labeled monoclonal antibody along with immunochromatography. The detected cryptococcal capsular polysaccharide antigen generates antigen–antibody binding reaction with colloidal gold-labeled antibodies. The antigen–antibody complex migrates to the surface of the test strip under capillary action while binding to the monoclonal antibody on the nitrocellulose membrane, which leads to the appearance of a red line, which is known as the test line [15]. A negative control is not required, since the test strip itself contains a control line; thus, the result is more reliable.

BALF is performed by injecting normal saline into the bronchial alveoli through a bronchoscope, which is followed by the aspiration and collection of the alveolar surface lining fluid to analyze its soluble components. This technique has become the main investigative method for the diagnosis of pulmonary disorders. The BALF technique has advantages, such as convenience, time-efficiency, reduced trauma, and high sensitivity. Relevant studies have revealed that the sensitivity, specificity, and positive predictive value of the BALF GM test are significantly higher than those of serum for the diagnosis of invasive pulmonary aspergillosis [3, 16, 17]. Hence, this study compared the results of performing CrAg-LFA in BALF and serum and revealed that the sensitivity and accuracy of BALF CrAg-LFA for PC diagnosis were superior to those of serum CrAg-LFA and microbial culture (93.9%, 98.2%; 72.7%, 91.0%; 27.3%, and 78.4%, respectively) with a statistically significant difference (*p* < 0.05). Furthermore, previous studies revealed that the overall sensitivity and specificity of serum CrAg-LFA for the diagnosis of cryptococcal infection were 97.6% and 98.1%, respectively [15]. However, the sensitivity for the same was lower in this study than that in the previous literature, since previous studies mostly focused on patients with HIV-positive cryptococcal meningitis. This study revealed a superior performance of BALF CrAg-LFA in PC diagnosis for the following two reasons in addition to the accurate localization of the BALF collection site by high-resolution chest CT: (1) less fluid is required for CrAg-LFA, which avoids the dilution of the samples caused by the excessive perfusion fluid, which in turn affects the positivity rate of the test results. A small amount of perfusion fluid (20–30 mL only) was used to accurately collect the lavage samples of the target bronchus to raise the positive rate in this study. (2) If there was no growth of fungal colonies when the *Cryptococcus* culture time was prolonged to 2 weeks, the culture was considered negative in this study, thereby reducing the missed diagnosis rate. Since *Cryptococcus* is an aerobic organism, it has a high requirement for a growth environment (Cap) and takes a long time to culture. In the absence of HIV infection in cases with PC, a culture result was obtained for BALF within at least a week. However, the positive rate was approximately only 20% [18].

Previous studies have highlighted the limitation of *Cryptococcus* antigen as reduced sensitivity in PC diagnosis at the early stage of infection or in isolated foci [19]. Only 56% of patients with non-HIV PC infection showed positive serum *Cryptococcus* antigen on the agglutination test [15, 20]. In this study, of the nine patients with PC who were pathologically diagnosed as serum CrAg-LFA negative, six were BALF CrAg-LFA-positive. This indicated that the positive rate and sensitivity of BALF CrAg-LFA were higher than that of serum, which may be related to local infiltrative [2] *Cryptococcus* growth in the lung, high *Cryptococcus* load in the lesion area, and no or reduced release of *Cryptococcus* antigen into blood in the early stage of the infection. Additionally, in this study, the lesion in one patient was located in the right upper lobe anterior segment, who was CrAg-LFA-negative for serum and the whole right upper lobe bronchial wash fluid, whereas the patient was CrAg-LFA-positive for the lavage fluid collected from flushing the targeted subsegmental bronchus, thereby, suggesting that a positive result from flushing fluid of the lesion tissue can only be obtained in the early stage of the infection. In this study, only two cases were confirmed to be CrAg-LFA-negative both in serum and BALF. The findings were observed by physical examinations and these cases did not have clinical symptoms. However, there were small peripheral isolated lesions located in the anterior basal and inner basal segments of the lower lobe of the right lung. Therefore, BALF CrAg-LFA may also produce false-negative results for cases with small peripheral lesions in the absence of clinical symptoms and early sickness, and pathological diagnosis may be selected if necessary. Additionally, no patient showed positive serum CrAg-LFA but had negative BALF CrAg-LFA.

The colloidal gold method might also generate false-positive results in CrAg detection [21, 22]. In this study, serum CrAg-LFA was weakly positive, but BALF CrAg-LFA was negative in 1 patient with sicca syndrome (1.3%) of the 78 patients with non-PC, suggesting that BALF CrAg-LFA was more accurate than serum CrAg-LFA. Therefore, in the case where the serum test results are weakly positive in clinical settings, taking into account the potential cross-reaction of serum and antigens in diseases, reexamination with semiquantitative assay or bronchoscopic BALF is suggested to reduce the chances of misdiagnosis in cases of weakly positive serum test results. Additionally, the chance of a potential cross-reaction of the serum *Cryptococcus* antigen between sicca syndrome and PC remains to be further explored. In this study, no false-positive results were found in any of the BALF samples, and the assay specificity was 100%. The result of BALF CrAg-LFA might still be positive for patients with negative serum antigens, thus potentially avoiding unnecessary invasive procedures. Therefore, we recommend that lung puncture and surgery resection be considered only in cases of negative BALF CrAg-LFA.

However, this retrospective study has some limitations. First, the sample size was small and the subsequent treatment effects in patients were not followed up. Therefore, the efficacy of BALF CrAg-LFA for PC diagnosis and treatment should be further evaluated by increasing the sample size and follow-up duration. Second, the differences between BALF CrAg-LFA and traditional diagnostic methods (such as latex agglutination and ELISA) were not compared. Thus, differences among the various detection methods using BALF need to be further evaluated to provide more complete experimental data for clinical practice.

## 5. Conclusions

PC complexity, diversity, and concealment require vigilance from clinicians. The diagnostic value of BALF CrAg-LFA for PC is superior to that of serum CrAg-LFA and BALF etiological culture. BALF CrAg-LFA has the advantages of simplicity, speediness, safety, and accuracy, with an important clinical significance for early PC diagnosis inpatients.

## Figures and Tables

**Table 1:** Clinical characteristics of recruited participants.

Characteristics	PC group (*n* = 33), *n* (%)	Non-PC group (*n* = 78), *n* (%)	*P* value
Mean age (years)	40.3 ± 12.46	53.5 ± 17.13	0.679
Gender			
Male	23 (69.7)	43 (55.1)	0.153
Female	10 (30.3)	35 (44.9)	
Smoking status			
Smokers	6 (18.2)	24 (30.8)	0.172
Nonsmokers	27 (81.8)	54 (69.2)	
Clinical symptoms			
Fever (> 37.5°C)	7 (21.2)	14 (17.9)	0.688
Cough/sputum	17 (51.5)	31 (39.7)	0.253
Shortness of breath	6 (18.2)	21 (26.9)	0.327
Chest pain/chest distress	5 (15.2)	12 (15.4)	0.975
Hemoptysis	6 (18.2)	12 (15.4)	0.715
Asymptomatic	18 (54.5)	14 (17.9)	0.000
Dizziness/headache	6 (18.2)	7 (9.0)	0.168
Comorbidities			
Chronic bronchitis	3 (9.1)	6 (7.7)	1.000^a^
Diabetes mellitus	7 (21.2)	11 (14.1)	0.353
Cirrhosis	1 (3.0)	2 (2.6)	1.000^b^
Chronic kidney disease	1 (3.0)	5 (6.4)	0.794^a^
Malignant solid tumor	6 (18.2)	7 (9.0)	0.168
Autoimmune disease	1 (3.0)	5 (6.4)	0.794^a^
No underlying disease	10 (30.3)	11 (14.1)	0.046
Corticosteroids use (＞3 weeds)			
Inhaled glucocorticoid	1 (3.0)	5 (6.4)	0.794^a^
Systemic corticosteroids	0 (0.0)	3 (3.8)	0.553^b^
Immunosuppressive agent use	0 (0.0)	2 (2.6)	1.000^b^
White blood cells (×10^9^/l)	6.71 ± 1.93	7.33 ± 4.10	0.404
Neutrophil (%)	4.22 ± 1.65	4.13 ± 2.37	0.891
Lymphocytes (%)	20.01 ± 6.32	23.11 ± 9.52	0.917
C-reactive protein (mg/dl)	20.15 ± 22.03	27.34 ± 48.27	0.267
CD4^+^ T lymphocytes (%)	41.14 ± 10.70	40.4 ± 8.68	0.736
CD8^+^ T lymphocytes (%)	24.13 ± 11.17	24.66 ± 10.31	0.900
Chest CT Findings			
Single nodule/mass	9 (27.3)	13 (16.7)	0.846
Multiple nodules/masses	11 (33.3)	12 (15.4)	0.006
Mediastinal lymph node	0 (0.0)	8 (10.3)	0.102^b^
Pleural effusion	1 (3.0)	7 (9.0)	0.432^a^

^a^Chi-squared test with continuity correction; ^b^Fishers exact test; PC, pulmonary cryptococcosis; CT, computer tomography.

**Table 2:** Comparison of culture results of *Cryptococcus* in serum and BALF.

Group	*N*	Serum		BALF	
		Positive	Negative	Positive	Negative
PC	33	1 (3.0)	32 (97.0)	9 (27.3)	24 (72.7)
Non-PC	78	0 (0.0)	78 (100.0)	0 (0.0)	78 (100.0)
Sum	111	1	110	9	102
*χ* ^2^		30.03^*∗*^		22.04^*∗*^	
*P* value		<0.001		<0.001	

^*∗*^The McNemar test; BALF: bronchoalveolar lavage fluid: PC, pulmonary cryptococcosis.

**Table 3:** Comparison of the CrAg-LFA results in serum and BALF.

Group	*N*	Serum		BALF	
		Positive	Negative	Positive	Negative
PC	33	24 (72.7)	9 (27.3)	31 (93.9)	2 (6.1)
Non-PC	78	1 (1.3)	77 (98.7)	0 (0.0)	78 (100.0)
Sum	111	25	86	31	80
*χ* ^2^		4.90^*∗*^		0.50^*∗*^	
*P* value		0.021		0.049	

^*∗*^The McNemar test; CrAg-LFA, cryptococcal antigen-lateral flow assay.

**Table 4:** Diagnostic value of serum and BALF CrAg-LFA in PC (% (cases/total cases)).

Specimen type	Sensitivity	Specificity	Accuracy
Serum	72.7 (24/33)	98.7 (77/78)	91.0 (101/111)
BALF	93.9 (31/33)	100.0 (78/78)	98.2 (109/111)
*χ* ^2^	4.000^*∗*^	0.000^*∗*^	6.125
*P* value	0.039	1.000	0.008

^*∗*^The McNemar test.

**Table 5:** Diagnostic value of CrAg-LFA and culture for PC in BALF (% (cases/total cases)).

Diagnostic methods	Sensitivity	Specificity	Accuracy
Culture (*Cryptococcus*)	27.3 (9/33)	100.0 (78/78)	78.4 (87/111)
CrAg-LFA	93.9 (31/33)	100.0 (78/78)	98.2 (109/111)
*χ* ^2^	20.045^*∗*^	0.000^*∗*^	18.375
*P* value	<0.001	1.000	<0.001

^*∗*^The McNemar test.

## Data Availability

The datasets used and analyzed during the current study are available from the corresponding author upon request.
